# A “Broken Heart” syndrome case after cesarean delivery. Prevention and risk assessment.

**Published:** 2013-09-02

**Authors:** R. Petta, F. Zullo, A. Tipaldi, M. Guida

**Affiliations:** 1 Department of Gynecology and Obstetrics, University of Salerno, Salerno, Italy

A 35-year-old woman in the fortieth week of her first pregnancy was admitted into our Department for terminal pregnancy.

She underwent cesarian delivery after forty-eight hours, and eight hours later, she showed cephalea, growing dyspnea and cyanosis.

Pneumonologic and cardiac advice were required. Chest examination showed: damp sounds in the right upper lung zone. Heart examination showed: blood pressure of 95/70mmHg, medium heart rate of 86 beats/min (max heart rate: 129, min heart rate: 54).

Upon physical examination, the woman was afebrile with a body temperature of 36°C.

She was intubated and treated by intravenous inotropic therapy.

An arterial blood-gas analysis gave the following values: pH, 7.462; partial pressure of oxygen, 38.7 mmHg; carbon dioxide pressure, 26,3 mmHg; bicarbonate, 18.5 mmol/L.

Electrocardiography showed: sinus rhythm (86/min), ST-segment changes without necrosis and ischemia; supraventricular ectopic beats of 63SV; no bradycardia; no paroxysmal supraventricular tachycardia.

Echocardiography revealed: severe LV systolic dysfunction with akinesia of the LV base and mid-portion, together with hypercontractility of the apex and ejection fraction of 30%; mild mitral and tricuspid regurgitation; pulmonary hypertension with a pulmonary arterial pressure of 45 mmHg; no pericardium effusion; Inferior Vena Cava congestion.

Coronary angiography revealed that both coronary arteries were intact [ 1 ] .

Because of the patient’s hemodynamic instability, intra-aortic balloon pumping was begun, and medical treatment that included inotropic agents. The woman was admitted into Cardiac Intensive Care Unit [ 2 ] .

Laboratory findings included raised levels of: ferritine (506ng/ml; normal 10–291ng/ml), troponin I (1,73ng/ml; normal 0–0,10) and PCR (12,3 mg/dl; normal <0,5); CK muscle and brain (MB) is 63,1 ng/ml (reference range 10–92).

The patient received cardiogenic shock due to takotsubo cardiomyopathy treatment. She responded well to treatment: her hemodynamic state stabilized one day later; intra-aortic balloon pumping was discontinued two days later and tracheal tube was removed.

About two weeks later, echocardiography showed complete recovery of LV systolic and diastolic function, chest radiography was normal so the patient was discharged [ 3 ] .

This case further supports the importance of perforrming a pre-conception planning, and proper counselling about the timing and mode of delivery by a coordinated decision between the gynaecologist, anaesthesiologist and cardiologist to balances mother’s and fetus’s risks ( Table 1 , Table 2 ). The purpose is the early diagnosis and the best management of the cardiomyopathy [ 4 – 6 ] .

## Figures and Tables

**Figure 1:**
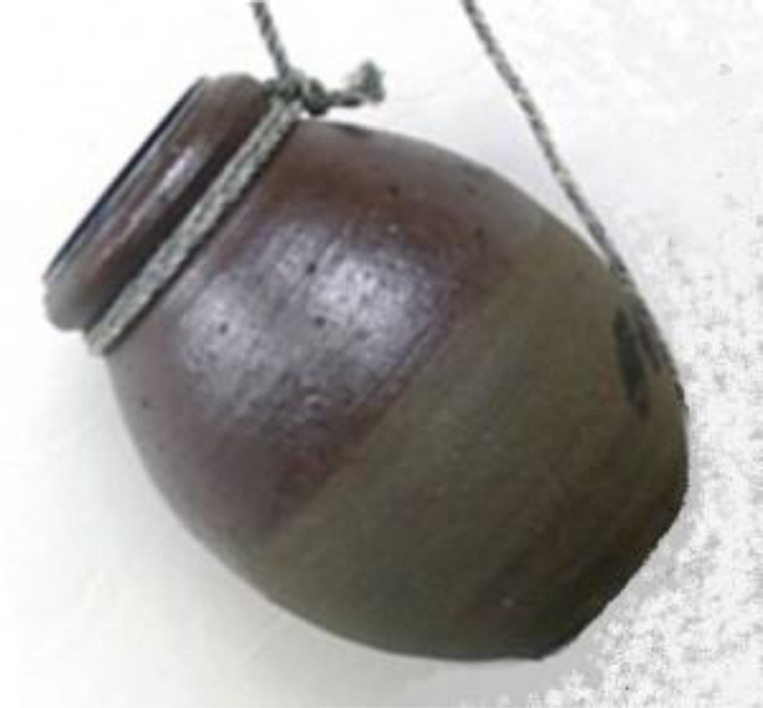
A picture of a real takotsubo, a Japanese octopus fishing pot.

**Figure 2:**
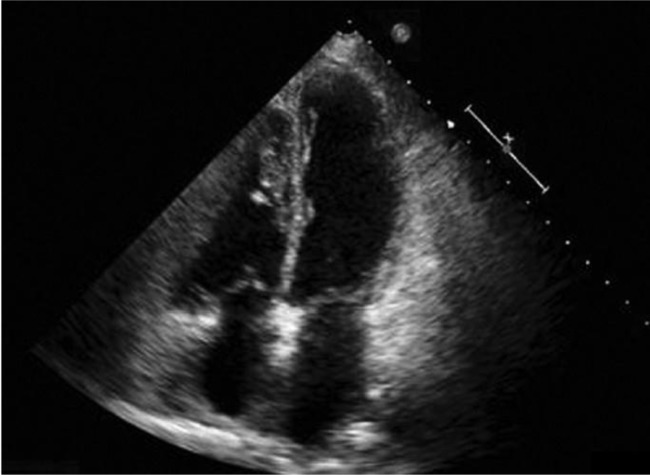
Echocardiography of takotsubo.

**Table 1:** Pregnancy after Tako Tsubo: risk assessment

	Pre-conception evaluation
-	Thorough history of cardiac symptoms and physical examination
-	12-lead electrocardiogram
-	Baseline exercise tolerance and functional class (exercise testing if needed)
-	Baseline echocardiogram
-	Assessment of ventricular function (right and left)
-	Assessment of pulmonary artery pressure
-	Presence and degree of valvular dysfunction
-	Assessment of stability of cardiac hemodynamic status over time
-	Effective contraception until pregnancy desired
-	Adjust medications to prevent adverse fetal events
-	Genetics referral for patients with heritable cardiac lesion

**Table 2:** Tako Tsubo prevention during labour and delivery

	Decision regarding timing and mode of delivery
-	Management during labor and delivery and post-partum concerns
-	Short vaginal delivery with excellent anesthesia, with consideration of assisted second stage of labor
-	Left lateral decubitus position
-	Cesarean section per obstetric indications
-	Invasive monitoring if needed (right heart catheterization, invasive arterial blood pressure monitoring)
-	Medical therapy optimization of loading conditions
-	Consider treatment of severe anemia
-	Medical therapy to optimize loading conditions
-	Hemodynamic monitoring for 12–24 h
-	Contraception or sterilization consideration
-	Future consideration of ICD

## References

[1] 
Parsad
 
S
 (
2007
). Apical ballooning syndrome: an important differential diagnosis of acute myocardial infarction. Circulation.

[2] 
Abe
 
Yoshiteru
, 
Kondo
 
Makoto
, 
Matsuoka
 
Ryota
, 
Araki
 
Makoto
, 
Dohyama
 
Kiyoshi
, 
Tanio
 
Hitoshi
 (
2003
). Assessment of Clinical Features in Transient Left Ventricular Apical Ballooning. J. Am. Coll. Cardiol.

[3] 
Lee
 
Sahng
, 
Jin Lee
 
Kyung
, 
Yoon
 
Hyeon Soo
, 
Kang
 
Ki Woon
, 
Lee
 
Young Sook
, 
Lee
 
Jun Wan
 (
2010
). Atypical Transient Stress-Induced Cardiomyopathies with an Inverted Takotsubo Pattern in Sepsis and in the Postpartal State. Tex Heart Inst J.

[4] 
Crimi
 
Ettore
, 
Baggish
 
Aaron
, 
Leffert
 
Lisa
, 
Pian-Smith
 
May CM
, 
Jannuzzi
 
James L
, 
Jang
 
Yandong
 (
2008
). Acute Reversible Stress-Induced Cardiomyopathy Associated with Cesarean Delivery under Spinal Anesthesia. Circulation.

[5] 
Yaqub
 
Yasir
, 
Jenkins
 
Leigh Ann
, 
Nugent
 
Kenneth M
, 
Chokesuwattanaskul
 
Warangkana
 (
2009
). Pastpartum Depression and Apical Ballooning Syndrome (Takotsubo Syndrome). J Obstet Gynaecol Can.

[6] 
Stergiopoulos
 
K
, 
Shiang
 
E
, 
Bench
 
T
 (
2011
). Pregnancy in patients with pre-existing cardiomyopathies. J Am Coll Cardiol.

